# The Effect of Membrane Surface Hydrophobicity on the Performance and Water Production Cost of a Desalination Unit

**DOI:** 10.3390/membranes15020063

**Published:** 2025-02-14

**Authors:** Sima Rabiei, Anthony H. J. Paterson

**Affiliations:** School of Food and Advanced Technology, Massey University, Palmerston North 4472, New Zealand; a.paterson@massey.ac.nz

**Keywords:** membrane distillation, membrane hydrophobicity, pore wetting, pore size, water production cost

## Abstract

Membrane pore wetting remains a significant challenge to achieving the stable operation and commercialization of membrane distillation processes. This study quantitatively assessed membrane surface hydrophobicity to investigate its impact on the performance and water production cost of an MD system. Membranes with a similar pore wetting resistance but differing in surface hydrophobicity and pore diameter were examined. A direct contact membrane distillation unit was modeled, and the water flux results were compared with laboratory experiments to validate the model. The validated model was subsequently employed to simulate a seawater desalination plant with a designed capacity of 20 m^3^/day. The results demonstrated that membranes with a higher surface hydrophobicity and bigger pore sizes achieved higher water flux, increasing from 0.6 kg/m^2^·h to 2.5 kg/m^2^·h, and significantly reduced water production costs from NZD$13.5/m^3^ to $3.9/m^3^. This research highlights the importance of optimizing membrane surface properties and microstructures to advance MD applications.

## 1. Introduction

Seawater desalination has emerged as a vital solution to address global water scarcity. With approximately 97% of the Earth’s water being saline, desalination offers significant potential for freshwater production [1]. Reverse osmosis (RO) remains the most commercially viable desalination technology, but its high energy demand has driven interest in developing alternatives that rely on renewable energy sources [2].

Membrane distillation (MD) as a novel membrane separation process utilizes a vapor pressure gradient across a hydrophobic microporous membrane to separate volatile molecules [3]. MD presents several advantages, including operation at low pressures and temperatures, the complete rejection of non-volatile solutes, and the capability to treat concentrates, such as RO brine, due to the absence of osmotic pressure limitations [4]. Furthermore, the low operating temperatures in MD processes enable the use of alternative energy sources, such as waste heat from industrial plants [5], geothermal energy [6], and solar energy [7]. These attributes make MD a promising technology for small-scale [8] and off-grid desalination applications [9].

Among MD configurations, direct contact membrane distillation (DCMD) is the simplest, involving direct contact between the hot feed and cold distillate with the membrane surfaces, where evaporation and condensation occur [10]. To prevent liquids from entering the membrane pores, the applied pressure should be kept below the liquid entry pressure (LEP) of the membrane [10]. The hydrophobic characteristic of the microporous membrane ideally prevents liquids from entering the pores, allowing only vapor and non-condensable gases to be present within the membrane pores [11].

Hydrophobic membrane can be fabricated in various configurations, such as single layer hydrophobic membranes, dual-layer composite membranes, or multi-layer structures. Polymeric membranes, like Polytetrafluoroethylene (PTFE), Polypropylene (PP), and Polyvinylidene fluoride (PVDF), are widely favored for MD due to their ease of fabrication, modification, and cost-effectiveness [11].

Research shows that enhancing membrane hydrophobicity can delay pore wetting and improve MD stability [12]. The hydrophobicity of a membrane can be enhanced either by chemical modification to lower the surface energy or by the introduction of proper surface roughness. The multi-level surface roughness provides grooves on the surface, and while air would be trapped within the grooves, water droplets cannot follow the surface contour. Superhydrophobic surfaces, characterized by contact angles exceeding 150°, minimize the direct contact between the water and the membrane by trapping air within the surface grooves [13].

Membranes with higher surface hydrophobicity also alter the heat exchange between liquids and the membrane surface. This alteration occurs due to the creation of an isolating air film adjacent to the membrane surface, generated by hydrophobic repulsions. The presence of this air layer reduces the thermal diffusivity and temperature polarization within the boundary layers [11].

In addition to surface hydrophobicity, the microstructure of the membrane, including the surface and bulk porosity, pore size, tortuosity, and thickness, plays a critical role in optimizing MD performance. Higher porosity and bigger pore sizes enhance permeability but may compromise membrane mechanical strength and increase the risk or membrane pore wetting, resulting in a trade-off between permeability and pore wetting prevention [14]. Thus, achieving a balance between permeability and process stability is essential for sustained performance.

Several cost analyses of MD systems, often integrated with low-grade energy sources or RO plants, have highlighted the economic potential of MD in specific scenarios. The MEDESOL project, aiming at the development of a stand-alone solar desalination system for production of 50 m^3^/day water using MD, analyzed the water production costs of different small solar systems. The estimated water production costs (WPCs) ranged from $15.6/m^3^ for brackish water to $31.3/m^3^ for seawater, depending on the operational conditions and configurations [15]. Other small-scale solar-driven MD systems achieved WPCs of between $5.16/m^3^ and $18.26/m^3^, depending on scale and design [16,17].

While numerous studies have investigated the relationship between membrane properties and surface wettability, a gap remains in understanding the extent to which membrane selection impacts the overall performance and economic feasibility of MD systems. This study addresses this gap by evaluating MD’s competitiveness, particularly in scenarios where waste energy can be utilized to power the system. Furthermore, a commercial assessment is incorporated, highlighting the critical role of membrane selection in optimizing MD processes for desalination.

A DCMD unit with a production capacity of 20 m^3^/day, driven by waste heat energy, was modeled and experimentally validated. This research aims to advance the understanding of membrane design and its role in enhancing MD competitiveness and economic feasibility, particularly in contexts where waste heat can be utilized as an energy source.

## 2. Materials and Methods

### 2.1. Desalination Plant

**Figure 1:**
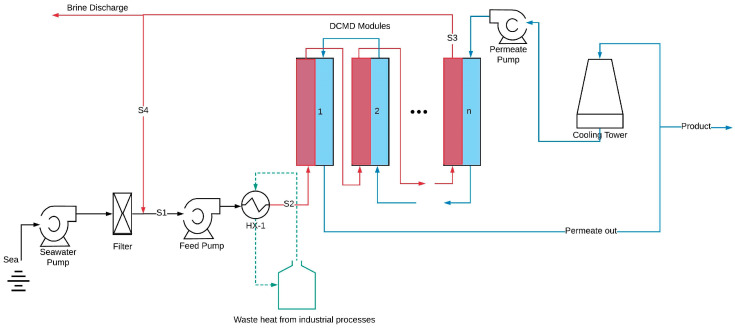
Schematic diagram of a hypothetical seawater desalination plant based on DCMD.

Figure 1 illustrates a schematic diagram of the hypothetical seawater desalination plant. The proposed plant operates in a constant recovery mode, where a portion of the concentrated brine (stream S3) is discharged from the system, while the remaining portion (stream S4) is recycled back into the process. This recycling process enhances thermal efficiency and increases water recovery [18].

The process begins with seawater, containing a salt concentration of 3.5 wt.% and an ambient temperature of 20 °C, passing through a filtration system to remove suspended solids. The filtered seawater is then mixed with the recycled brine stream (S4), resulting in an increase in both the temperature and salt concentration of the feed water.

The outlet feed stream from the MD modules is set to reach a salt concentration of 25% (*w*/*v*), which is maintained below the salt saturation limit of 35% at 20 °C to prevent salt crystallization within the system. This feed outlet concentration supports a water recovery ratio of 83%, calculated as the volumetric flow rate of the product water divided by the volumetric flow rate of the seawater entering the plant [19].

Stream S1 is directed into the HX-1 heat exchanger, where it is heated using available waste heat until reaching the desired feed inlet temperature (*T_f,in_*). Potential sources of waste heat include return cooling water from petrochemical industries, flue gases, or steam. However, specific details about the waste heat source are excluded to allow for a more generalized cost analysis. While this study assumes waste heat to be freely available, we acknowledge that heat sources above 80 °C may not always be accessible at no cost. However, as demonstrated by Jantaporn et al. [20], the specific energy requirement for DCMD is primarily dependent on the recovery factor, not membrane properties, so thermal energy consumption is excluded from the cost analysis, focusing instead on other cost-contributing factors.

Once heated, the feed enters the membrane distillation (MD) modules as stream S2 to achieve the target concentration. The cold stream is introduced into the MD modules in a counter-current flow configuration to maximize the temperature gradient, exiting at the opposite end. Afterwards, the cold flow undergoes further cooling via a cooling tower or pond.

Table 1 summarizes the membrane and module specifications used in this study. A plate-and-frame MD configuration was selected, as it enables higher flow rates, thereby enhancing water flux [21]. Each module is equipped with a suitable mesh spacer to promote fluid mixing and includes a total membrane area of 1.25 m^2^.

To improve channel length, membrane area, and hot stream residence time, the membrane modules are arranged in a combination of series and parallel configurations. However, increasing the channel length can reduce mass flux across the module due to a decreased driving force. Therefore, it is necessary to optimize the membrane length to achieve the best performance. In this study, the optimal configuration includes five membrane modules connected in series, resulting in a total membrane area of 6.25 m^2^ for each cascade with a counter-current flow.

### 2.2. Mathematical Modeling of DCMD

**Figure 2:**
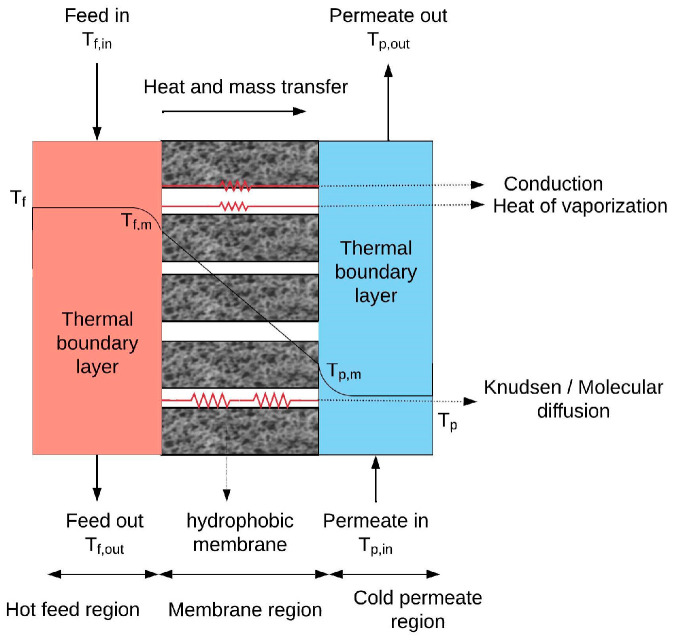
Schematic of the heat and mass transfer processes in DCMD.

To evaluate the effects of membrane surface hydrophobicity and pore size on the performance of DCMD, a comprehensive model was developed to simulate the coupled heat and mass transfer processes. Figure 2 provides a schematic representation of the DCMD process operating in a counter-current configuration [22].

The transmembrane mass flux *J* (kg·m^−2^·s^−1^) is described by the equation below:

(1) $$J=C(p_{f,m}-p_{p,m})$$

The MD coefficient *C* (kg·m^−2^·s^−1^·Pa^−1^) depends on various factors, such as membrane characteristics, vapor properties, and membrane surface temperatures [23]. The water vapor pressures at the membrane surfaces on the feed and permeate sides, denoted as *p_f,m_* (Pa) and *p_p,m_*, respectively, are calculated using the Antoine equation based on their corresponding surface temperatures [22]:

(2) $$p=\exp(23.328-\frac{3841}{T-45})$$

where *T* (°K) and *p* (Pa) are the membrane surface temperature and water vapor pressure, respectively. The water vapor pressure at the feed side, *p_f,m_*, is lowered due to the presence of dissolved salt, with the mole fraction of *x_s,f_*, as described by Raoult’s law, which is expressed by the following Equation (3) [24]:

(3) $$p_{f,m}=p_{f,m}^{w}(1-x_{s,f})$$

where $p_{f,m}^{w}$ (Pa) is the vapor pressure for pure water with no dissolved species.

The mean pore size of the membrane plays a significant role in determining the mass transfer mechanisms through the porous membrane [25]. The Knudsen number (*Kn*), as given by Equation (4), is a crucial parameter that determines the dominant mass transfer mechanism through the pores [23]:

*Kn* = λ/*dp* (4)

where *d_p_* (m) is the membrane pore diameter, and λ (m) is the mean free path of gas molecules across the pores. Depending on the value of *Kn*, the mass transfer through the pores can be dominated by transition, Knudsen diffusion, or molecular diffusion mechanisms. Specifically, for *Kn* values less than 0.1, the mass transfer is dominated by convective flow and molecular diffusion in the transition regime. For *Kn* values between 0.1 and 10, Knudsen diffusion, characterized by gas molecules bouncing between pore walls, becomes the dominant mass transfer mechanism. For *Kn* values of greater than ten, molecular diffusion, with gas molecules diffusing through the pores without any collisions, dominates the mass transfer process [26]. For membranes featuring pore sizes smaller than 0.5 μm, a typical pore diameter for membranes in MD systems, the combined Knudsen/molecular diffusion model is typically applied, and Equation (5) is used to determine the MD coefficient [26,27]:

(5) C=32τδεrπRT¯8Mw12+τδεPaPDw−aRT¯Mw−1

Wherein, the parameters *r* (m), *δ* (m), *τ*, and *ε* represent the average pore radius, thickness, tortuosity, and porosity of the membrane, respectively. *M_w_* (kg·kmol^−1^) denotes the molecular weight of water, *D_w-a_* (m^2^·s^−1^) signifies the water vapor diffusivity in air, and *R* (J·kmol^−1^·K^−1^) represents the gas constant. *P_a_* (Pa) and *P* (Pa) are the air pressure and total pressure in the membrane pore, respectively. Meanwhile, $\bar{T\;}$(°K) represents the membrane mean temperature, which can be obtained from Equation (6) [26]:

(6) $$\bar{T}=\frac{T_{f,m}+T_{p,m}}{2}$$

where *T_p,m_* and *T_f,m_* (°K) are the surface temperatures of the membrane at the permeate flow and feed sides, respectively. *D_w-a_* is determined by Equation (7) [23]:

(7) $$PD_{w-a}=1.895\times 10^{-5}{\bar{T}}^{2.072}$$

The membrane distillation coefficient, as described by Equation (5), is significantly influenced by the properties of the membrane, including pore size, porosity, thickness, and tortuosity. Among these properties, thickness and pore size are likely to have a more dominant effect on permeation, as porosity and tortuosity tend to fall within a narrow range [14].

In a steady state, heat transfer in DCMD involves three main components: convective heat flux through the feed boundary layer (*Q_f_*), the combined conductive heat flux and latent heat associated with mass flux through the membrane (*Q_m_*), and convective heat flux through the permeate flow boundary layer (*Q_p_*). The mathematical expressions for these heat transfers are presented in Equations (8)–(10) [28]:

(8) $$Q_{f}=h_{f}\;(T_{f}-T_{f,m})$$

(9) $$Q_{m}=\frac{k_{m}}{\delta }\left(T_{f,m}-T_{p,m}\right)+J{∆H}_{v}\;(\bar{T})$$

(10) $$Q_{p}=h_{p}\;(T_{p,m}-T_{p})$$

The heat transfer coefficients on the permeate flow and feed sides, denoted as *h_p_* and *h_f_* (W·m^−2^·K^−1^), respectively, are calculated using appropriate empirical correlations. *T_p_* and *T_f_* (°K) represent the average temperatures of the permeate flow and feed bulk, respectively.

The vaporization enthalpy at the membrane mean temperature is denoted as Δ*H_v_* (J·kg^−1^) and can be obtained from [29].

The membrane thermal conductivity, *k_m_* (W·m^−1^·K^−1^), is determined using Equation (11), which involves the thermal conductivities of the gas in the pores, $k_{g},$ and the membrane polymer, *k_p_*, respectively [30]:

(11) $$k_{m}=\left(1-\varepsilon \right)k_{p}+\varepsilon k_{g}$$

The conductive heat transfer through the membrane is considered as heat loss. Equation (12) is utilized to determine the thermal efficiency (*η*) of the MD process, which represents the fraction of the total heat transferred through the membrane that is associated with the mass flux [10]:

(12) $$\eta =\frac{J{∆H}_{\nu }}{\frac{k_{m}}{\delta }\left(T_{f,m}-T_{p,m}\right)+J{∆H}_{\nu }}$$

By considering the overall heat balance in the steady state ($Q_{p}{=Q_{m}=Q}_{f}$), the surface temperatures of the membrane can be calculated using Equations (13) and (14) [4]:

(13) $$T_{f,m}=\frac{\frac{k_{m}}{\delta }\left(T_{p}+(\frac{h_{f}}{h_{p}})T_{f}\right)+h_{f}T_{f}-J{∆H}_{\nu }}{\frac{k_{m}}{\delta }\left(1+\frac{h_{f}}{h_{p}}\right)+h_{f}}$$

(14) $$T_{p,m}=\frac{\frac{k_{m}}{\delta }\left(T_{f}+(\frac{h_{p}}{h_{f}})T_{p}\right)+h_{p}T_{p}+J{∆H}_{\nu }}{\frac{k_{m}}{\delta }\left(1+\frac{h_{p}}{h_{f}}\right)+h_{p}}$$

The temperature of the membrane surfaces deviates from the bulk temperatures, which is referred to as temperature polarization, and the temperature polarization coefficient (*TPC*) can be determined using Equation (15) [31]:

(15) $$TPC=\frac{T_{f,m}-T_{p,m}}{T_{f}-T_{p}}$$

The heat transfer coefficients *h_f_* and *h_p_* are determined using appropriate empirical correlations based on the flow regime (laminar or turbulent) within the membrane channel, as described in Equations (16)–(18) [30]:

(16) $$Nu=0.13{Re}^{0.64}{Pr}^{0.38}\;\;Re<2100$$

(17) $$Nu=0.023\;{Re}^{0.8}{Pr}^{0.33}\;\;Re>2100$$

(18) $$h=\frac{Nu\cdot k}{D_{h}}$$

where *Pr*, *Re*, and *Nu* represent the Prandtl, Reynolds, and Nusselt numbers, respectively, and *k* (W·m^−1^·K^−1^) and *D_h_* (m) denote the liquid thermal conductivity and the hydraulic diameter of the module, respectively.

### 2.3. Membrane Pore Wetting

To prevent pore wetting and maintain selectivity during MD operation, it is essential to ensure that the LEP of the membrane remains higher than the applied pressure. The LEP can be calculated using the Laplace (Cantor) equation, which is expressed as follows [27]:

(19) $$LEP=\frac{-2B\gamma_{l}cos\theta }{r_{max}}>{∆P}_{interface}=P_{liquid}-P_{vapor}$$

where B, $r_{max}\;$(m), *θ* (deg.), and *γ*_l_ (N·m^−1^) represent the pore geometric factor, the maximum membrane pore size, the liquid contact angle with the membrane surface, and the surface tension of the liquid, respectively. The average pore size of the membrane was assumed to be half of the maximum membrane pore size to investigate the impact of the hydrophobic property of the membrane surface on the performance of the DCMD.

### 2.4. Code Development

**Figure 3:**
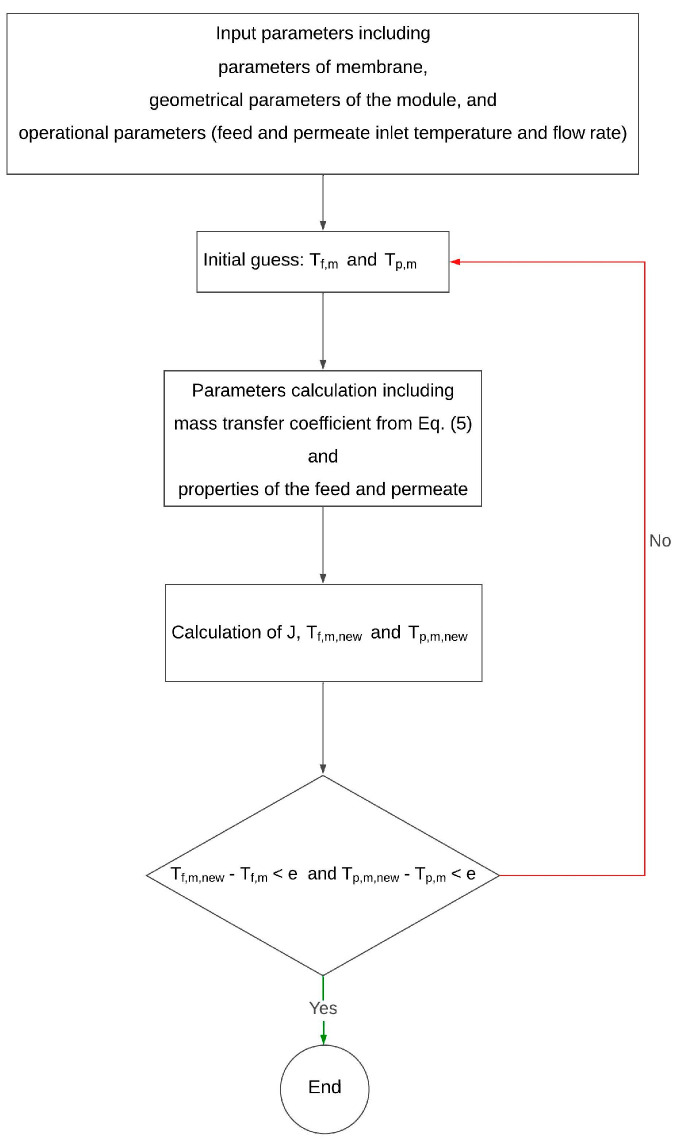
Algorithm for the iterative calculation of DCMD water flux.

The DCMD model was developed using Visual Basic in conjugation with Microsoft Excel, as shown in Figure 3. The model begins by defining membrane morphological parameters, module geometry, and operating conditions. An iterative approach calculates water flux, starting with initial guesses for the membrane surface temperatures on both sides, initially assumed to be the bulk temperatures of the feed and permeate. The water flux is then used to update the membrane surface temperatures. This process iterates until the difference between consecutive membrane surface temperature values is less than 10^−7^ K, indicating convergence. Theoretical water flux predictions are compared with experimental results to validate the model’s accuracy.

### 2.5. DCMD Experiments

**Figure 4:**
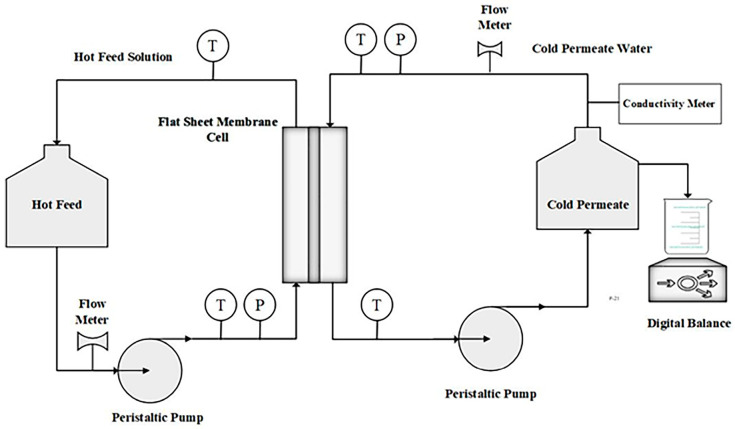
Schematic diagram of the experimental setup for DCMD used in this study.

The experimental validation of the model was conducted under varying feed inlet temperatures. A schematic representation of the DCMD process is provided in Figure 4, with additional details on the experimental setup available in [32]. The membrane used was a PVDF microporous membrane (Hangzhou Tianshan Precision Filter Material Co., Ltd., Hangzhou, China) with a mean pore diameter of 0.22 μm and a thickness of 110 μm, as determined by the SEM analysis. The membrane porosity, determined using the gravimetric method described in [32], was 71 ± 1.5%.

The feed solution contained 3.5 wt.% NaCl. The water flux was measured by recording the weight of the collected permeate over a 2 h period. Experiments were conducted at different feed inlet temperatures, ranging from 60 to 80 °C, with a constant feed flow velocity of 0.11 m/s. Each experiment was repeated ten times under identical conditions, with flux variations observed within ±5%.

### 2.6. Cost Calculation

The water production cost (WPC) of a desalination plant is influenced by various factors, including plant capacity, lifespan, feed water quality, pretreatment requirements, process technology, and energy costs [33]. In this study, the WPC for the proposed desalination plant was estimated based on the total capital costs (TCCs), which included equipment, instrumentation, and control costs. Additionally, annual operating costs, such as energy consumption and membrane replacement, were considered [26,33,34]. Land costs were excluded from the calculations, as MD systems typically require a small footprint. The data and assumptions used in the WPC calculations are summarized in Table 2.

The cost of the hydrophobic PVDF membranes used in this study was assumed to be $60/m^2^, which is comparable to the reported cost of PTFE membranes in previous research [35]. The membrane cost was considered to constitute 33.3% of the total cost of the membrane module, including both the cost of membrane itself and its assembly. Therefore, each MD module, with a membrane area of 1.25 m^2^, was estimated to cost $225, based on the criteria outlined by Banat and Jwaied [33].

The purchase cost of pumps was determined based on the required shaft power and referring to the cost curve for cast iron centrifugal pumps found in the literature [36]. Similarly, the purchase cost of the heat exchanger was calculated by considering the required heat transfer area, derived from simulation results, and referring to a specific cost curve tailored for fixed tube sheet heat exchangers with a carbon steel shell and tube design [36]. To account for inflation, the estimated costs of pumps and the heat exchanger were adjusted to the Chemical Engineering Plant Cost Index (CEPCI) of 1982, then converted to the year 2019 using the most recent CEPCI available at the time of the analysis. The membrane cost was estimated based on the required membrane area, determined from the plant’s production capacity and the DCMD water flux. The annualized capital costs ($/year) were calculated by multiplying the total capital costs (TCCs) by the amortization factor presented in Equation (20), assuming a 5% interest rate for borrowed funds [33].

(20) $$a=\frac{i{\left(1+i\right)}^{n}}{{\left(1+i\right)}^{n}-1}$$

where *i* (%/year) represents the annual interest rate, and *n* (years) represents the expected plant lifetime.

The yearly operating cost in this study is calculated based on the annual energy consumption, with an assumption of 20% membrane replacement per year. In MD plants, the required thermal energy is a major contributor to the WPC, as stated in previous studies [35]. To meet this thermal energy requirement, it is assumed that any available waste energy is utilized. This is commonly feasible in industrial plants that generate cooling or hot streams as discharge, as suggested in [26]. Therefore, the energy requirement in this study is primarily limited to the electricity consumption for pumping, $E_{p}$, which can be calculated using Equation (21):

(21) $$E_{p}=\frac{q_{v}∆P}{\eta }$$

where *q_v_* (m^3^/s) and Δ*P* (Pa) represent the flow rate and the pressure difference in the pump, respectively. The pump efficiency, denoted by η, is assumed to be 65%. The desalination plant is assumed to operate continuously throughout the year. To calculate the annual electricity cost ($/yr.), the electricity consumption is multiplied by the specific electricity cost ($/kWh) and the total number of operating hours per year (24 h/day × 365 days/year × plant availability factor).

The *WPC* ($/m^3^_product_) is then determined by adding the amortized *annual capital cost* ($/yr.) and the *annual operating costs* ($/yr.) and dividing the sum by the *annual production* (m^3^_product_/yr.), as expressed in Equation (22):

(22) $$WPC=\frac{\left(Annual\;capital\;costs+yearly\;operating\;costs\right)}{Annual\;production}$$

## 3. Results and Discussions

### 3.1. Model Validation

**Figure 5:**
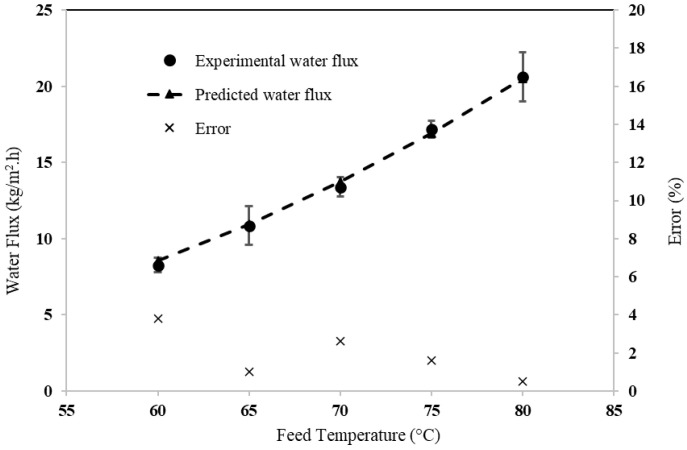
Assessment of modeling at different feed temperatures (feed and permeate flow velocities = 0.11 m·s^−1^; cold inlet temperature = 20 °C).

Figure 5 compares the predicted and measured water flux at different feed inlet temperatures, ranging from 60 to 80 °C. The results show close agreement between the predicted and experimental data, with a deviation of less than 5%. Any discrepancies observed between the predicted and experimental results are likely due to factors such as the membrane’s microstructure and pore interconnectivity, which were not included in the model. Previous studies have indicated that DCMD flux increases with higher feed temperatures due to an enhanced driving force, which is consistent with the model’s predictions. These findings suggest that the model used in this study accurately predicts the performance of the DCMD system.

### 3.2. Effect of Membrane Characteristics on LEP

**Figure 6:**
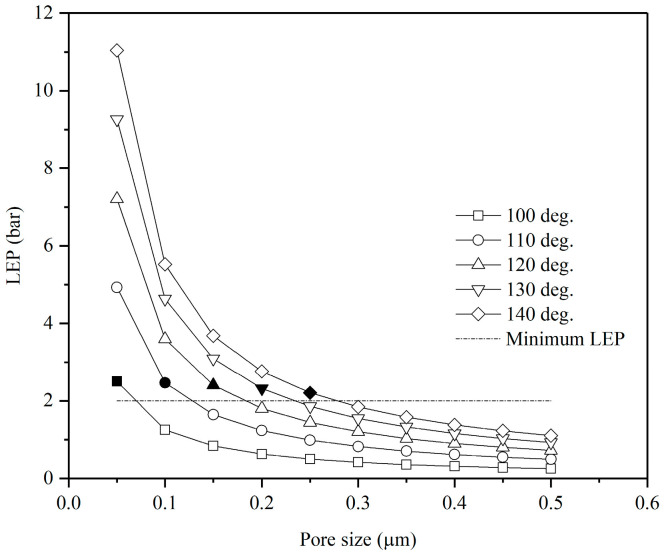
Influence of average membrane pore size on LEP at varying surface contact angles.

Figure 6 presents the liquid entry pressure (LEP) values calculated using the Laplace equation for membranes with varying pore sizes (0.05 to 0.5 μm) and surface contact angles (100° to 140°). According to this figure, bigger pore sizes result in lower LEP values, making membranes more susceptible to pore wetting. Conversely, membranes with higher surface contact angles exhibit greater resistance to pore wetting.

According to the Laplace equation, highly hydrophobic and superhydrophobic membranes enable increased pore size while effectively preventing pore wetting, thereby enhancing membrane permeability and improving MD performance [37]. A previous study indicates that membranes with LEP values of below 2 bars are more prone to wetting during long-term operation [38]. Therefore, this study focuses on membranes with LEP values exceeding two bars, as represented by the black data points.

This correlation between membrane surface hydrophobicity and microstructure properties provides a valuable insight into assessing DCMD performance and the associated costs, considering pore wetting as a critical factor.

### 3.3. Effect of Membrane Hydrophobicity on DCMD Flux

**Figure 7:**
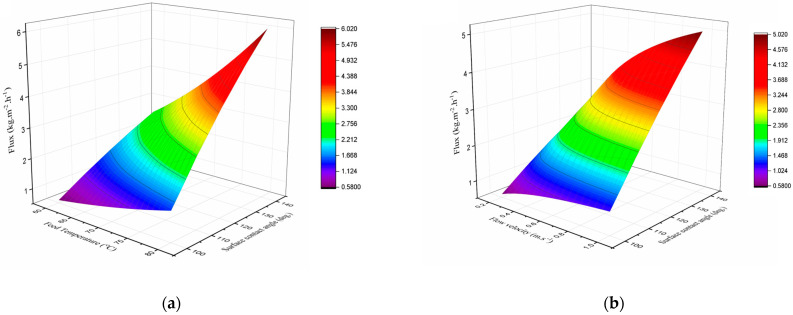
Effect of operating parameters and membrane characteristics on DCMD water flux: (**a**) feed inlet temperature, ranging from 60 to 80 °C at constant flow velocity of 0.2 m/s; (**b**) feed and permeate flow velocities, ranging from 0.2 to 1 m/s at constant feed inlet temperature of 60 °C.

Figure 7 presents surface plots showing the simulated water flux of the DCMD unit for membranes with varying surface contact angles and pore sizes under different operating conditions. In Figure 7a, the flow velocity for both the feed and permeate streams is fixed at 0.2 m/s, while the feed inlet temperature increases from 60 °C to 80 °C. The contours show an exponential rise in water flux with the increasing feed inlet temperature. This behavior is attributed to the exponential increase in the water vapor pressure gradient across the membrane, as described by the Antoine equation.

Although operating MD systems at elevated feed temperatures is beneficial, moderate feed temperatures are more practical when using low-grade waste heat as the thermal energy source.

In Figure 7b, the effect of feed and permeate flow velocities, ranging from 0.2 to 1 m/s, on the DCMD unit water flux is illustrated, with the feed inlet temperature held constant at 60 °C. The observed increase in water flux with higher flow velocities is attributed to enhanced hydrodynamic conditions at higher Reynolds numbers. These conditions suppress boundary layer resistance and reduce the impacts of temperature and concentration polarization. However, at higher flow velocities, the water flux reaches a plateau, becoming less sensitive to any further increase in flow velocity. This plateau occurs because the thermal boundary layer thickness decreases with increasing flow velocity until it becomes independent of further changes in velocity [11].

As illustrated in Figure 7a,b, under specific operating conditions, the DCMD water flux demonstrates an almost linear increase with membranes that exhibit higher surface hydrophobicity and larger pore sizes. At the reference point, with a feed inlet temperature of 60 °C and flow velocities of 0.2 m/s, replacing a membrane with a surface contact angle of 100° with one featuring a contact angle of 140° resulted in an increase in water flux by up to 4.2 times.

It is also noteworthy that membranes with greater hydrophobicity not only improve water flux but also demonstrate enhanced resistance to pore wetting, potentially extending the operational lifespan of the membranes.

### 3.4. Effect of Membrane Hydrophobicity on DCMD Thermal Efficiency

**Figure 8:**
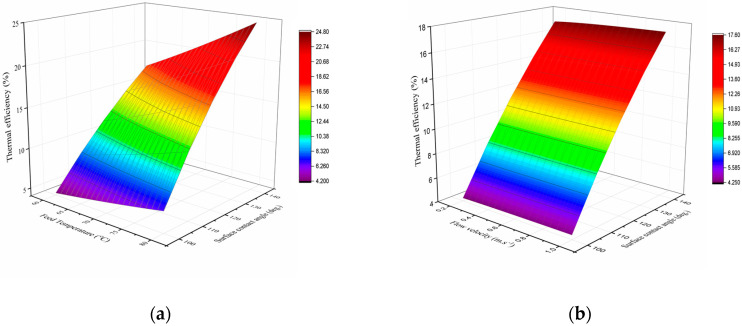
Effect of operating parameters and membrane characteristics on DCMD thermal efficiency: (**a**) feed inlet temperature, ranging from 60 to 80 °C at a constant flow velocity of 0.2 m/s; (**b**) feed and permeate flow velocities, ranging from 0.2 to 1 m/s at constant feed inlet temperature of 60 °C.

Thermal efficiency is defined as the ratio of the latent heat of vaporization to the total heat, including both latent and conductive heat, passing through the membrane. Figure 8 presents the simulation results for the thermal efficiency of the DCMD unit as a function of membrane surface hydrophobicity and operating parameters.

Figure 8a illustrates that increasing the feed temperature from 60 to 80 °C improves the thermal efficiency almost linearly. This increase is due to the enhanced driving force for mass transfer as the feed temperature rises, which amplifies the effect of the latent heat. On the other hand, Figure 8b shows that increasing the feed and permeate flow velocities leads to a slight enhancement in thermal efficiency. This can be attributed to the reduction in boundary layer resistance, facilitating heat transfer.

As discussed in Section 3.3, increasing the surface hydrophobicity of the membrane, moving towards a contact angle of 140°, results in a significant improvement in water flux. Consequently, this enhancement in water flux also contributes to an increase in thermal efficiency, as more heat is effectively utilized for vaporization, rather than lost to conduction.

At the reference point (with a feed inlet temperature and a flow velocity of 60 °C and 0.2 m/s, respectively), the thermal efficiency improves from 4.3% to 17.1% when a membrane with a higher surface contact angle and larger pore sizes is used. These findings highlight the significant role of membrane pore size in membrane distillation processes. By enhancing the surface hydrophobicity, it becomes feasible to utilize membranes with larger pore sizes, which, in turn, improves thermal efficiency while maintaining stable operations.

In addition to the direct effect of larger pore sizes on thermal efficiency, highly hydrophobic membranes also exert an indirect influence on the thermal efficiency of MD processes. The hydrophobicity reduces pore wetting and minimizes heat loss due to conduction, further enhancing the overall efficiency of the system. Therefore, optimizing both the membrane surface hydrophobicity and the pore size is essential for improving the performance of DCMD systems, balancing both water flux and thermal efficiency.

**Figure 9:**
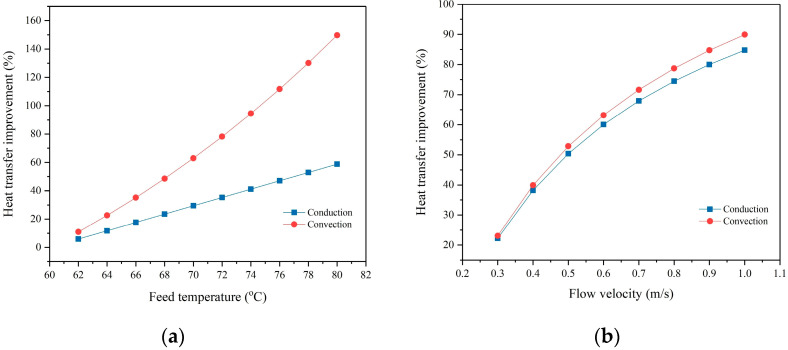
Effect of (**a**) feed inlet temperature and (**b**) flow velocity on the conductive and convective heat transfer through the DCMD process.

Figure 9 illustrates the impact of operational parameters on the thermal efficiency of the process, specifically focusing on the conductive and convective heat transfer contributions from the feed to the permeate side. As shown in Figure 9a, increasing the feed inlet temperature improves convective heat transfer due to the exponential rise in water vapor pressure with temperature. Additionally, higher feed temperatures also result in a linear increase in conductive heat transfer, which corresponds to heat loss, because of the increased transmembrane temperature difference. However, the rate of improvement in convective heat transfer is greater than that in conductive heat transfer, leading to a net increase in the thermal efficiency of the unit.

Figure 9b demonstrates the effect of feed and permeate flow velocities on both conductive and convective heat transfer contributions. As previously discussed, higher flow velocities in both the feed and permeate channels lead to increased water flux, which, in turn, increases the latent heat associated with convective heat transfer. Furthermore, higher flow velocities improve hydrodynamic conditions, which reduce the effects of temperature polarization and bring the surface temperatures of the membrane closer to the bulk temperatures of the feed and permeate. This improvement in hydrodynamic conditions contributes to increased conductive heat transfer due to the enhanced transmembrane temperature difference. Figure 9b shows that both convective and conductive heat transfers improve at a similar rate, with only a slight enhancement in thermal efficiency being observed as the flow velocities increase.

### 3.5. Effect of Membrane Hydrophobicity on Water Production Cost (WPC)

**Figure 10:**
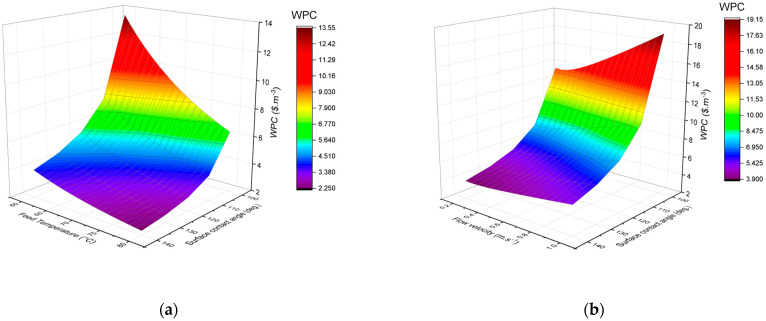
Effect of operating parameters and membrane characteristics on WPC: (**a**) feed inlet temperature, ranging from 60 to 80 °C at a constant flow velocity of 0.2 m/s; (**b**) feed and permeate flow velocities ranging from 0.2 to 1 m/s at a constant feed inlet temperature of 60 °C.

Figure 10 presents the variation in the WPC of the proposed plant under different operating conditions and varying membrane hydrophobicity, with values ranging from $2.25/m^3^ to $19.12/m^3^. Figure 10a demonstrates the impact of feed inlet temperature on the WPC, showing that higher feed temperatures lead to lower WPCs due to the associated improvements in water flux and thermal efficiency, leading to reduced capital costs. Figure 10b illustrates the effect of feed and permeate flow velocities on the WPC, which decreases to a minimum at a flow velocity of 0.25 m·s^−1^.

Beyond this threshold, the increased electricity consumption due to higher pumping requirements outweighs the benefits of the enhanced water flux, resulting in a higher WPC. Therefore, operating DCMD at a moderate flow velocity is preferable for achieving a reasonable balance between water flux and pumping costs.

**Figure 11:**
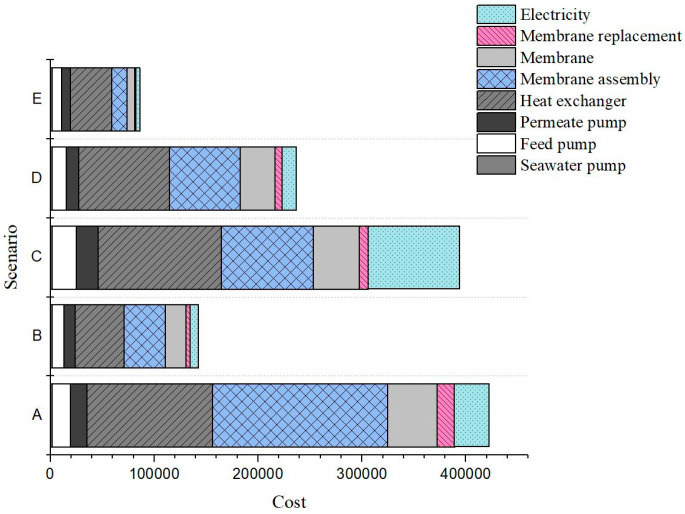
The distribution of capital and operating costs for the desalination plant under different operating conditions and varying membrane surface hydrophobicity.

Figure 11 shows a comprehensive summary of the cost analysis under various operating conditions and membrane hydrophobicity. The figure visually presents the portion of each cost item, as outlined in Table 3, which provides a detailed breakdown of the cost items and their primary contributors. This comparison enables a comprehensive understanding of the cost implications associated with different membrane and operating conditions. According to Figure 11, membrane costs remain a significant contributor to the overall WPC, followed by electricity costs.

The results in Table 3 highlight the sensitivity of water production costs (WPCs) to membrane hydrophobicity and operating parameters. Notably, increasing the membrane surface contact angle from 100° to 140° under constant operating conditions (scenarios A to B) leads to substantial reductions in the WPC from $13.51/m^3^ to $3.95/m^3^ due to the higher water flux and smaller system size. This is particularly evident in the reduction of membrane and membrane assembly costs, as the improved hydrophobicity minimizes the need for larger membrane surface areas.

Furthermore, optimizing the feed inlet temperature and flow velocity (scenario E) significantly affects both the thermal efficiency and operational costs. As the feed temperature increases from 60 °C to 80 °C, with a constant flow velocity and using the same membrane (scenarios A to D), the WPC decreases from $13.51/m^3^ to $6.37/m^3^ due to the enhanced thermal efficiency, which reduces the capital and operational costs, including membrane and assembly expenses. The most hydrophobic membrane, operating at the highest feed temperature and a low to moderate flow velocity, achieved the lowest WPC of $2.25/m^3^. However, the flow velocities above 0.25 m/s (scenarios A to C) exhibited diminishing returns, as the increase in energy consumption outweighs the benefits from higher water flux. This emphasizes the importance of finding a balance between flow velocity and energy consumption to achieve optimal economic performance.

For large-scale applications, optimizing membrane hydrophobicity, feed temperature, and flow velocity can yield competitive water production costs compared to other desalination technologies, like reverse osmosis (11.7–15.6 $/m^3^) and electrodialysis (10.4–11.7 $/m^3^ [39]. While heat was assumed to be totally free in this study, the results remain promising, as the costs would decrease in a large-scale plant due to cheaper membranes and module systems. Furthermore, the proposed desalination system, with a high water recovery ratio of 83%, can integrate the brine stream leaving the plant into a crystallization process for further salt recovery, leading to zero-discharge technology.

This study’s results emphasize that highly hydrophobic membranes with larger pore sizes and open microstructures enhance process continuity and improve water production costs. With appropriate adaptations, these insights can be extended to other MD setups, contributing to the broader application of MD in desalination and wastewater treatment. These findings suggest that membrane distillation (MD) powered by waste energy could become a viable, cost-effective, and environmentally sustainable alternative for large-scale desalination projects.

## 4. Conclusions

This research highlights that the development of highly hydrophobic membranes with suitable pore wetting resistance and permeability can significantly enhance the sustainability and economic viability of large-scale MD applications. The study stablishes a quantitative framework for the development of specialized membranes with improved hydrophobicity and enlarged pore structures to address key challenges in MD technology, such as pore wetting and low water flux. Focusing on membranes with sufficient pore wetting resistance, varying surface hydrophobicity, and average pore sizes, the results demonstrate that membranes with higher hydrophobicity and larger pores show increased permeability, leading to enhanced water production rates and a substantial 70% reduction in water production costs, from $13.51/m^3^ to $3.95/m^3^. Additionally, the study found that raising the feed inlet temperature from 60 to 80 °C resulted in a 53% reduction in WPC, although this cost reduction was less significant compared to the membrane-related improvements. The findings suggest that employing hydrophobic membranes with larger pore sizes, while ensuring adequate pore wetting resistance, can significantly lower the WPC, making membrane distillation a competitive option compared to other desalination technologies powered by waste energy at similar production capacities.

## Figures and Tables

**Table 1:** Characteristics of the membrane and module used in the desalination plant.

Property	Value	Unit
Membrane	PVDF	-
Membrane porosity (ε)	75%	-
Membrane thickness (δ)	100	μm
Channel dimensions (L × W × H)	0.5 × 0.5 × 0.004	m
The hydraulic diameter of the channel	0.008	m
The surface area of a membrane sheet	0.25	m^2^
Number of sheets per module	5	
Number of modules in series	5	

**Table 2:** Summary of data and assumptions for WPC calculations.

Item	Value	Unit	Reference
Plant Capacity	20	m^3^_product_/day	-
Plant Lifespan	20	years	[4]
Plant Availability Factor	90	%	[4]
Interest Rate	5	%	[4]
Electricity Cost	0.07	$/kWh	[35]
DCMD Module Cost	225	$/unit	-
Membrane Cost	60	$/m^2^	[35]
Membrane Replacement Rate	20	%/year	[35]

**Table 3:** Summary of cost items for selected operating conditions and membrane surface hydrophobicity.

Cost Items	*T_f_*_,*in*_ = 60 °C, u = 0.2 m/s, θ = 100° (A)	*T_f,in_* = 60 °C, u = 0.2 m/s, θ = 140° (B)	*T_f,in_* = 60 °C, u = 1 m/s, θ = 100° (C)	*T_f,in_* = 80 °C, u = 0.2 m/s, θ = 100° (D)	*T_f,in_* = 80 °C, u = 0.25 m/s, θ = 140° (E)
Heat exchanger ($)	121,254	47,214	118,697	87,348	39,770
Feed pump ($)	17,885	11,862	23,555	13,777	9492
Permeate pump ($)	15,738	10,434	20,743	12,148	8361
Seawater pump ($)	1465	1465	1465	1465	1465
Membrane ($)	84,005	19,857	44,228	33,890	7309
Membrane assembly ($)	168,010	39,714	88,456	67,780	14,618
Electricity ($/yr.)	33,475	7924	88,058	13,439	3633
Membrane replacement ($/yr.)	16,801	3971	8845	6778	1462
Annualized capital ($/yr.)	5.78	2.06	4.29	3.22	1.40
Annualized O&M ($/yr.)	7.72	1.88	14.82	3.15	0.85
WPC ($/m^3^)	13.51	3.95	19.12	6.37	2.25

## Data Availability

The data presented in this study are available upon request from the corresponding author. Access may be granted on a case-by-case basis due to privacy considerations.

## Abbreviations

The following symbols and abbreviations are used in this manuscript:

*A*	area [m^2^]	Greek letters	
*B*	pore geometric coefficient [-]	*γ*	liquid surface tension [N·m^−1^]
*C*	membrane distillation coefficient [g·m^−2^·s^−1^·Pa^−1^]	*δ*	thickness [m]
*D*	diffusivity [m^2^·s^−1^]	Δ*H*	latent heat [J·kg^−1^]
*d*	diameter [m]	*ε*	membrane porosity [-]
*d_h_*	hydraulic diameter [m]	*η*	efficiency
*H*	flow channel height [m]	*θ*	contact angle [degree]
*h*	heat transfer coefficient [W·m^−2^·K^−1^]	*λ*	mean free path [m]
*J*	permeate flux [kg·m^−2^·s^−1^]	*μ*	viscosity [N·s·m^−2^]
*K_B_*	Boltzmann constant [J·K^−1^]	*ρ*	density [kg·m^−3^]
*k*	thermal conductivity [W·m^−1^·K^−1^]	*σ*	molecular size [m]
*K**n*	Knudsen number [-]	*τ*	tortuosity of membrane [-]
*L*	flow channel length [m]	Subscripts	
LEP	liquid entry pressure [N·m^−2^]	*a*	air
*M*	molecular weight [kg·mol^−1^]	*c*	contact angle
*N**u*	Nusselt number [-]	*D*	molecular diffusion
$\dot{m}$	mass flow rate [kg·s^−1^]	*f*	feed
*P*	pressure [N·m^−2^]	*g*	gas
*p*	vapor pressure [N·m^−2^]	*i*	inlet
*Pr*	Prandtl number [-]	*k*	thermal conductivity [W·m^−1^·K^−1^]
*Q*	heat flux [W·m^−2^]	*K*	Knudsen diffusion
*q*	volumetric flow rate [m^3^·s^−1^]	*m*	membrane
*R*	gas constant [J·mol^−1^·K^−1^]	*o*	outlet
*Re*	Reynolds number [-]	*p*	permeate, pore
*r*	pore size [m]	*s*	salt
*T*	temperature [K]	*v*	vapor
*ʋ*	velocity [m·s^−1^]	*w*	water
*W*	flow channel width [m]		
*x*	solution concentration [-]		
Acronyms			
CEPCI	Chemical Engineering Plant Cost Index	RO	reverse osmosis
DCMD	direct contact membrane distillation	TCC	total capital cost
LEP	liquid entry pressure	TPC	temperature polarization coefficient
MD	membrane distillation	WPC	water production cost
